# Using the Internet to Support Exercise and Diet: A Stratified Norwegian Survey

**DOI:** 10.2196/med20.4116

**Published:** 2015-08-26

**Authors:** Silje C Wangberg, Tove Sørensen, Hege K Andreassen

**Affiliations:** ^1^ Department of Health and Society Narvik University College Narvik Norway; ^2^ Regional Centre on Substance Use Department of Addictions and Specialized Psychiatry University Hospital of North Norway Narvik Norway; ^3^ Norwegian Centre for Integrated Care and Telemedicine University Hospital of North Norway Tromsø Norway

**Keywords:** Internet, Health Behaviors, Social Disparities, Health Literacy

## Abstract

**Background:**

Internet is used for a variety of health related purposes. Use differs and has differential effects on health according to socioeconomic status.

**Objective:**

We investigated to what extent the Norwegian population use the Internet to support exercise and diet, what kind of services they use, and whether there are social disparities in use. We expected to find differences according to educational attainment.

**Methods:**

In November 2013 we surveyed a stratified sample of 2196 persons drawn from a Web panel of about 50,000 Norwegians over 15 years of age. The questionnaire included questions about using the Internet, including social network sites (SNS), or mobile apps in relation to exercise or diet, as well as background information about education, body image, and health. The survey email was opened by 1187 respondents (54%). Of these, 89 did not click on the survey hyperlink (declined to participate), while another 70 did not complete the survey. The final sample size is thus 1028 (87% response rate). Compared to the Norwegian census the sample had a slight under-representation of respondents under the age of 30 and with low education. The data was weighted accordingly before analyses.

**Results:**

Sixty-nine percent of women and 53% of men had read about exercise or diet on the Internet (χ^2^= 25.6, *P*<.001). More people with higher education (71%, χ^2^=19.1, *P*<.001), reported this. The same gender difference was found for using Internet-based interventions with 20% of women compared to14% of men reporting having used these interventions (χ^2^=7.9, *P=* .005), for having posted a status about exercise or diet on Facebook or other SNS (23% vs 12%, χ^2^=18.8, *P*<.001), and for having kept an online exercise or diet journal (21% vs 15%, χ^2^=7.0, *P*=.008). Evaluations of own physical appearance accounted for some of the gender differences in using online exercise or diet journals. Seven percent of the total sample reported having used electronic communication to ask professionals about exercise or diet, while a few more had discussed online with peers (10%). Asking professionals online was more common amongst those with only primary education (13%, χ^2^<10.5, *P*=.005).

**Conclusions:**

Gender and education are related to how the Internet is used to support health behaviors. We should be aware of the potential role of the Internet in accelerating social disparities in health, and continue to monitor population use. For Internet- and mobile-based interventions to support health behaviors, this study provides information relevant to tailoring of delivery media and components to user.

## Introduction

Currently, 85% of the Norwegian population use the Internet on an average day (1). Closing the access gap, however, makes socioeconomic differences in the use of Internet even more visible.

The concept “digital divide” is most often used to describe the gap between those who have access to computers and/or the Internet and those who have not (2). An important contribution of this concept is that it brought awareness of social inequalities into the reigning optimism with regards to the empowering potential of the Internet. There are, however, also some problems with this concept (2). One is that it makes the issue of access seem more dichotomous than it is. It obscures for instance, that quite a few people without access, especially older people, have others look up health information for them, or that health personnel and journalists use the Internet to a greater extent, also potentially influencing public health. Another issue obscured by this concept is that despite having access to the Internet, people engage in health related activities in it to a different extent, in different ways, and with different outcomes. Other concepts have been proposed that allows for more ambiguity while still pointing to the social inequalities, such as DiMaggio and colleagues’ (3) concept “digital inequalities”.

Pre-Internet concepts such as “health literacy” (4) may still be adequate for digital health purposes. “Health Literacy” is the skills needed by the individual to gain access to, understand, and use information in ways that promote and maintain good health (4). Nutbeam (4) has divided health literacy into three types: (1) functional literacy, which denotes sufficient basic skills in reading and writing to be able to function effectively in everyday situations; (2) interactive literacy, which denotes more advanced cognitive skills which, together with social skills, can be used to actively participate in, extract information from, and derive meaning from different forms of communication, and to apply new information to changing circumstances; and (3) critical literacy, which denotes cognitive and social skill to critically analyze information, and to use this information to exert greater control over life events and situations, such as engaging in shared decision making.

Some research groups have made even finer distinctions than this, but sufficient to say that the skills needed for successfully filling the roles of the active or empowered patient are diverse. Of course, doing all of this mediated via a computer and the Internet poses additional literacy demands on the user, both in terms of confidence and skills. This is covered by the concept “eHealth literacy”, first used by Norman and Skinner (5).

The social gradient in health is a well-established finding (6). Differences in education accounts for a substantial part of social disparities in health (7). One reason is that people with higher education are better able to attain and utilize health information to be proactive in relation to their own health (8,9). This can also be seen with regards to using the Internet for health purposes. People with higher education use the Internet more for finding health information (10,11).

Many have been optimistic about the potentials for the Internet to enhance both the efficiency and reach of health communication (12,13). The Internet has been used to deliver interventions for health behavior change, such as smoking cessation (14), diet (15,16) and physical activity (17). Internet-based interventions are in general slightly less effective than the face-to-face individual counseling alternatives, but has been found to be efficacious, as long as they are based on sound theory and existing knowledge about effective components in behavioral change interventions (18,19). Even a small effect size can make a substantial public health impact given large enough reach (20).

For more than a decade the Norwegian population has been surveyed on their general use of the Internet for health purposes. In 2007, 40% of the 67% having used the Internet for health purposes, reported that they had been inspired to change lifestyle as a result of Internet use, and 44% reported having searched for information about health behavior such as diet and smoking cessation (11). In the 2011/12 US “Health Information National Trends Survey” (HINTS), 43% of US adults reported having “used a website to help with diet, weight, or physical activity” during the last 12 months (21,22). Health behavior related use was reported by a significant lower proportion of respondents with a lower educational attainment, but by equal proportions of men and women (21,22).

These two and other eHealth surveys have established that there are educational differences in the use of Internet to support health behavior change. We ask in the current study if there are further differences in how the Internet is used for health promoting purposes according to educational attainment. From a vantage point of conceptualizing education-related differences in health information seeking as a continuum from non-seeking and avoidance, via passive and unsystematic information intake, to active, systematic and critical review of health information (23), we expected to find differences in Internet use according to educational attainment.

## Methods

### Design

A survey on use of the Internet for health-related purposes was conducted amongst a representative sample of Norwegian Internet-users in October and November 2013.

### Sample

A stratified sample of 2196 persons was drawn from TNS Gallup’s ISO 26362 certified Web panel of about 50,000 Norwegians over 15 years of age. The survey email was opened by 1187 respondents (54%). Of these, 89 did not click on the survey hyperlink (declined to participate), while another 70 did not return a completed survey. The final sample size for the current study is thus N=1028 (87% response rate of those contacted). The final sample had a slight underrepresentation of respondents under the age of 30 and a slightly higher educational attainment than the general Norwegian population according to census.

### Questionnaire

Collected sociodemographic information included gender, age, and educational attainment. Highest completed educational attainment was categorized as completed primary, secondary, or tertiary education. Lower secondary education (Norwegian “ungdomsskole”) was grouped with primary education and higher secondary education (Norwegian “videregående”) is referred to as secondary education. A completed Bachelors degree or its equivalent at college- or university-level, or higher is classified as tertiary education. In Norway, primary and lower secondary education is compulsory (normally completed during 6-16 years of age), while everyone has the right to three years of public higher secondary education (including vocational studies). Entrance to higher (tertiary) education is based on competition, but the public and tuition-free offer is extensive.

Subjective health was measured with the item: “How is your health in general? Would you say it is: 5 = “very good”, 4 = “good”, 3 = “fair”, 2 = “bad”, or 1 = “very bad”? To assess satisfaction with looks of own body, we used three items from the ”Appearance Evaluation Scale” (AES), which is a subscale of ”The Multidimensional Body-Self Relations Questionnaire” (MBSRQ). We used a Norwegian translation validated by Loland (24): (1) “I like my look the way it is”, (2) “Most people think I look good”, (3) “I like my looks without clothes”, all ranged on a five-point scale from 1 = “completely disagree” to 5 = “completely agree”. This is a widely used instrument, and the subscale we used has been shown to function similarly across gender and age groups (25).

Various Internet use for supporting health behavior was assessed with yes/no-questions such as: “do you have any experience of using the Internet or your mobile phone for any of the following:” (1) “reading about diet or physical activity”, (2) “posting a status update about diet or exercise on Facebook or other social network sites”, (3) “asking professionals a question about diet or exercise”, (4) “used an Internet- or mobile-based self-help program, that is, a service that provides help and guidance in changing a health behavior (such as diet, exercise or smoking). For some of the questions, like the last one (4) and “have you ever used a health app”, there is probably some overlap (for a complete list of questions, see Multimedia Appendix 1).

### Statistical Analyses

The data was weighted according to age and educational attainment to be representative of the general Norwegian population according to census. “I don’t know”-responses were counted as missing data and excluded from analyses in a pairwise fashion. None of the variables had more than 5% missing data. Dichotomous variables were made for education and subjective health for some of the analyses. A sum score ranging from 0-15 was computed for the appearance evaluation scale. Chi-square and ANOVA were used to test for differences between groups and logistic regression to analyze relationships between variables. Analyses were performed with IBM SPSS 19-22.

## Results

In the final weighted sample (N=1028), there were 50% men and 50% women. 27% of the men and 29% of the women had completed higher education (χ^2^=4.1, *P*=.044). Among those with a higher educational attainment 81% (241/299) report “good” or “very good” health compared with 69% (494/721) among those with secondary schooling or less (χ^2^=15.3, *P*<.001). The mean appearance evaluation score for men was 10.8 and 10.0 for women (F_1,1021_=18.1, *P*<.001), and those with a higher education (mean 10.9) was more satisfied with their looks than those with a lower education (mean 10.2, F_2,1021_=13.9, *P*<.001). There was no significant interaction between gender and education regarding appearance evaluation (F_2,1021_=1.2, *P*=.279).

Overall, 78% of the respondents reported some kind of health-related use of the Internet. The most commonly reported activity, by 61% of the respondents, was reading about exercise or diet on the Internet. Use of Internet- or mobile-based programs to support exercise or diet was reported by 17%. See Table 1 and 2 for the percentages of type of Internet-use related to diet or exercise stratified by gender and educational attainment.

If we look closer at gender differences first (Table 1), we find that more women than men reported having read about exercise or diet online (69% vs 53%), having used an Internet- or mobile-based program to support exercise or diet (20% vs 14%), having posted a status update about exercise or diet (23% vs 12%), or having kept an online exercise or diet journal (21% vs 15%). On the other hand, more men (9%) than women (7%) reported having shared online exercise or diet data with others (χ^2^=13.4, *P*<.001). There were no gender differences in the frequency of having asked professionals questions about exercise or diet (7%), or having discussed exercise or diet with peers (10%).

**Table 1:** Respondents’ online health behavior by gender.

	Total	Women	Men	Test statistics	
	n (%)	n (%)	n (%)	χ^2^	*P* value
Read about exercise or diet	614/1003 (61.22)	347/503 (68.99)	267/500 (53.40)	25.6	<.001
Asked questions about exercise or diet to professionals	68/1012 (6.72)	34/505 (6.73)	34/507 (6.71)	0.001	.987
Discussed exercise or diet with peers	97/1003 (9.67)	49/503 (9.74)	48/500 (9.60)	0.006	.940
Used Internet- or mobile-based programs to support health behavior	170/1006 (16.90)	102/505 (20.20)	68/501 (13.57)	7.9	.005
Posted a status about exercise or diet on Facebook or other SNS^a^	175/1003 (17.45)	114/504 (22.62)	61/499 (12.22)	18.8	<.001
Kept an online exercise or diet journal	180/1007 (17.87)	106/503 (21.07)	74/504 (14.68)	7.0	.008
Shared online exercise or diet data with others	76/1007 (7.55)	33/503 (6.56)	43/504 (8.53)	13.4	<.001

^a^SNS: social network site

As for differences in use of the Internet for supporting health exercise or diet with according to educational attainment (Table 2), more people with higher educational attainment had read about exercise or diet (71% vs 62% and 56%), posted a status about exercise or diet (23% vs 14% and 16%), or kept and online exercise or diet journal (25% vs 16% and 15%). On the other hand, those with only primary education had to a greater extent used the Internet to ask professionals questions about exercise or diet (13% vs 2% and 6%).

**Table 2:** Respondents’ online health behavior by educational attainment.

	Primary	Secondary	Tertiary	Test statistics	
	n (%)	n (%)	n (%)	χ^2^	*P* value
Read about exercise or diet	90/146 (61.6)	310/556 (55.8)	213/300 (71.0)	19.1	<.001
Asked questions about exercise or diet to professionals	19/150 (12.7)	13/561 (2.3)	18/300 (6.0)	10.5	.005
Discussed exercise or diet with peers	20/148 (13.5)	50/507 (9.9)	27/271 (10.0)	2.94	.230
Used Internet- or mobile-based programs to support health behavior	29/147 (19.7)	88/561 (15.7)	53/299 (17.7)	1.57	.456
Posted a status about exercise or diet on Facebook or other SNS^a^	21/149 (14.1)	87/558 (15.6)	68/297 (22.9)	8.58	<.014
Kept an online exercise or diet journal	23/148 (15.5)	82/558 (14.7)	75/300 (25.0)	14.8	001
Shared online exercise or diet data with others	9/148 (6.1)	36/558 (6.5)	30/300 (10.0)	0.26	.876

^a^SNS = Social Network Site

A two-block logistic regression was performed with having kept an online exercise or diet journal as the dependent variable and education, gender, subjective health as the independent variables in step one and additionally appearance evaluation in step 2. The final model can be seen in Table 3 and accounted for 3-4% of the explained variance in having kept an online exercise or diet journal. In the first step, when controlling for each other, being female, having a higher education and a good or very good subjective health were all positively related to having kept an online exercise or diet journal. In block two, when we added appearance evaluation, we see that it reduced gender to non-significance, suggesting that some of the relation between gender and having kept an online exercise or diet journal can be explained by appearance evaluation.

**Table 3:** Logistic regression with having kept an online exercise or diet diary as dependent outcome (N=1002).

Block	Independent variable	Odds ratio (95%CI)	*P*
1	Good or very good subjective health	1.71 (1.15-2.59)	.009
	Higher education	1.76 (1.26-2.47)	.001
	Female	1.57 (1.13-2.18)	.008
2	Good or very good subjective health	1.92 (1.23-3.00)	.004
	Higher education	1.69 (1.18-2.44)	.005
	Female	1.40 (0.98-1.99)	.067
	More satisfied with appearance	0.96 (0.90-1.03)	.262

## Discussion

### Principal Findings

Most Norwegians have access to the Internet, and close to 80% of the population use it for some kind of health related purpose, most commonly reading about exercise or diet, reported by 61%. Of special interest to those of us who develop such interventions, 17% of Norwegians in this survey had used an Internet- or mobile-based program to support exercise or diet. This finding justifies the often-stated reach potential of Internet- and mobile-based interventions.

While several other kinds of health behavior related use of the Internet were unequally distributed in the population, the use of self-help programs did not differ according to educational attainment. However, women, use these interventions more frequently than men do.

A more specific question in our survey concerned the keeping of an online exercise or diet journal. There were 25% of those with higher education who reported keeping such journals, compared to 15-16% among those with a lower educational attainment. This is an important finding because self-monitoring is one of the most effective behavior change techniques we know of (26). Thus, it seems that those with a higher educational attainment use the Internet to support health behavior change in more effective ways.

As in previous research, we found that those with higher education had read about exercise or diet online to a greater extent than those with lower education. Zillien and Hargittai (27) found that of those with the highest socioeconomic status (SES), 45% reported searching for health information, compared to 29% in the lowest SES group, and 40% across all seven status groups. The status effect on health related Internet use did, however, become insignificant when controlling for age, gender and interest in topic, all which were significant predictors of health related Internet use (27). The finding that interest in health is a moderator between SES and health related Internet use is not surprising from a functionalistic account of media use, as people with higher SES are expected to utilize the Internet to meet their needs, whether the topic is politics, stocks or health.

In addition to a greater health information orientation (9), people with high educational attainment also have higher Internet information processing skills (28). Birru and colleagues (29) showed some of the problems encountered by low-literacy adults when trying to find health information online. The participants had problems specifying effective search terms, tended to prefer sponsored links (which lead to alternative cancer treatments), and often ended up in websites with a high readability level. Many of the participants could identify and read back the relevant information on these sites, but were unable to paraphrase the information in their own words, suggesting limited comprehension of the material. In light of this, it is understandable that those with only a primary education in our survey to a greater extent had used the Internet to ask professionals about exercise or diet, a strategy which is likely to prove more effective than embarking on their own search.

We previously suggested that one of the ways that Internet might contribute to accelerating socioeconomic differences in health is through enabling those with a higher socioeconomic status to gain more health resources, including social support (30). In the current study, we found no significant differences between education groups with regards to discussing exercise or diet with peers online. Posting status updates about exercise or diet on Facebook or other social network sites was, however, more common among those with a higher educational attainment. This suggests that we need to refine our hypothesis and differentiate between different kinds of social support with regards to SES and health. Perhaps those with a lower educational attainment can utilize the Internet to gain instrumental support, while those with the highest educational attainment to a greater extent utilize the Internet to maintain a large social network. Furthermore, it is likely that posting statuses about exercise and diet could serve as a social class marker (31).

We, as other researchers, found that women are generally more active eHealth users (22,32). This finding has been explained in various ways, with women being the family’s health liaisons, poorer subjective health status, and reproductive issues as popular explanations. However, many of these suggestions have been more or less refuted in research on consultation rates with general practitioners (33). Although this study focused on the use of the Internet or mobile phones for monitoring the individual’s own health behavior, we still found that women were the more active eHealth users. This indicates that we need additional explanations for gender variance. With regards to women and health behaviors it has been suggested that it might have as much to do with appearance as with health (34). We found that as for monitoring one’s own exercise or diet in the form of keeping an online diary, controlling for appearance evaluation did reduce the effect of gender to non-significance, supporting that this may indeed be some of the explanation for women being more motivated to use eHealth tools to support their health behavior.

A weakness of our study is that we did not ask about watching videos online. Health literacy increase with the use of pictures, especially for those with a lower educational attainment (35). Previous studies of media preference (eg, 36) also found that TV were preferred over text by those with a lower educational attainment. Furthermore, some of the items we did ask about are probably overlapping, and not very precisely defined in terms of probably catching some eHealth use that is not directly related to health behaviors. We decided on this strategy of using and reporting several potentially overlapping items to make sure that we did not underestimate frequency of use based on the participant not being familiar with what we chose to name the activity, for example, “Internet-based intervention” would probably not ring any bells with most participants.

Another weakness of our study is that we lack detailed information about the participant’s health behavior and whether there were any changes after Internet use. Future research could employ longitudinal designs that incorporate observational measures of health behaviors and critical health literacy to elucidate more of the causal relationship between education level and health outcomes as mediated via using the Internet.

Empowerment and health literacy are necessary first steps in health promotion. This means that in order to utilize the Internet in health promoting ways, a person would need to: (1) be able to read, write and technically use the Internet, (2) have an internal locus of control (37) with regards to own health, and sufficient self-efficacy (38) for health behaviors that there seems a point to seek out health information or sign up for interventions, and (3) abilities to critically analyze and apply health information in a way that promotes own health. Offering eHealth services in lieu of measures to improve health literacy and sense of control in relation to personal health, will thus only benefit those who have gained these prerequisites themselves, and hence further empower those on the “winning end” of the social gradient.

There are at least two approaches we can take to accommodate the knowledge about social disparities in health behaviors into Internet-based interventions. We suggest that we first start with a screening of (e)health literacy (39-41) and then for those scoring under a certain threshold offer (1) a pre-intervention to increase (e)health literacy (eg, 42, 43) and/or (2) offer an intervention that rely more on interaction with professionals and/or peers and using more multi-media-based edutainment (23,35).

### Conclusions

Gender and education are related to how the Internet is used to support health behaviors. Women and people with higher education are more likely to use the Internet to support their health behaviors. However, men are more interested in uploading and sharing diet or exercise data, and people with lower education use the Internet more for communicating with others about diet or exercise. It becomes more and more evident that just providing universal access to eHealth in itself will only perpetuate and probably accelerate current social disparities in health.

## Appendix

Multimedia Appendix 1 Survey questions concerning different kinds of exercise and diet related use of the Internet.
