# Supplementary material for: Using the Internet to Support Exercise and Diet: A Stratified Norwegian Survey
Source: Med 2 0. 2015 Aug 26;4(2):e3. doi: 10.2196/med20.4116 (PMC4705024; doi:10.2196/med20.4116)
Supplement: Multimedia Appendix 1 [file med20_v4i2e3_app1.pdf]

Appendix 1. Questions about Internet use for supporting health behaviors

Original Norwegian Wording	English translation
Lest om kosthold eller trening på Internett (også via mobil)?	Read about diet or exercise on the Internet (also via mobile phone)?
Brukt Internett, epost, sms og mobilapplikasjoner ('Apper') for å spørre fagfolk om kosthold eller trening?	Used the Internet, e-mail, short message service (SMS), or mobile applications (‘Apps’) to ask professionals about diet or exercise?
Brukt Internett, epost, sms og mobilapplikasjoner ('Apper') for å diskutere med andre (ikke fagfolk) som er opptatt av kosthold eller trening?	Used the Internet, e-mail, short message service (SMS), or mobile applications (‘Apps’) to discuss with others (non-professionals) who are concerned about diet or exercise?
Brukt Internett/mobilbaserte selvhjelpsprogram, dvs tjenester som gir deg råd og hjelp i forhold til å ivareta din helse?	Used Internet- or mobile-based self-help programs, i.e. services that provide advice and help with regards to manage your health?
Postet en statusoppdatering om trening eller kosthold på Facebook eller et annet nettsamfunn?	Posted a status update about exercise or diet on Facebook or on another social network site?
Ført trenings- eller kostholdsdagbok på Internett? Dette gjelder også om du har lastet opp data fra mobiltelefonen din eller en pulsklokke.	Kept an exercise or diet journal on the Internet? This includes having uploaded data from your mobile phone or a heart rate monitor.
Hvis ja, har du delt trenings- eller kostholdsdataene dine med andre?	If yes, have you shared your exercise or diet data with others?
